# Assessment of *Yam mild mosaic virus* coat protein gene sequence diversity reveals the prevalence of cosmopolitan and African group of isolates in Ghana and Nigeria

**DOI:** 10.1016/j.cpb.2020.100156

**Published:** 2020-09

**Authors:** Chukwuemeka K. Nkere, Emmanuel Otoo, Gabriel I. Atiri, Joseph Onyeka, Gonçalo Silva, Moritz Bömer, Susan E. Seal, P. Lava Kumar

**Affiliations:** a International Institute of Tropical Agriculture, PMB, 5320, Ibadan, Nigeria; b Department of Crop Protection and Environmental Biology, University of Ibadan, Nigeria; c National Root Crops Research Institute, Umudike, PMB, 7006, Umuahia, Nigeria; d CSIR-Crops Research Institute, Kumasi, P.O. Box 3785, Fumesua, Ghana; e Natural Resources Institute, University of Greenwich, Central Avenue, Chatham, ME4 4TB, UK

**Keywords:** YMMV, Yam mild mosaic virus, YMV, Yam mosaic virus, Yam, *Dioscorea* spp., Virus, Ghana, Nigeria

## Abstract

This study analyzed the genetic diversity of 18 Yam mild mosaic virus (YMMV, genus Potyvirus) isolates collected from field surveys in Ghana (N = 8) and Nigeria (N = 10) in 2012−13. The full coat protein (CP) encoding region of the virus genome was sequenced and used for comparison and phylogenetic analysis of the YMMV isolates available in the NCBI nucleotide database. The mean nucleotide (nt) diversity was 13.4% among the 18 isolates (17 from *D. alata* and one from *D. rotundata*), 11.4% within the isolates of Ghana and 7.4% within the isolates of Nigeria. The phylogenetic clustering of the 18 YMMV isolates did not show correlation with the country of origin, and they aligned with the reference sequences of four of the 11 YMMV monophyletic groups representing the cosmopolitan group and the African group of YMMV isolates. High sequence homology of 99% between the YMMV sequence from Nigeria (CP12-DaN6-1) and a previously reported sequence from Togo (GenBank Accession Number AF548514) suggests a prevalence of seed-borne virus spread within the region. Understanding YMMV sequence diversity in West Africa aid in the improvement of diagnostic assays necessary for virus indexing and seed certification.

## Highlights

• Sequence diversity show lack of geographical association between isolates from Ghana and Nigeria.
• YMMV isolates from Ghana and Nigeria fall within four of the 11 monophyletic groups.
• Need for stringent control of germplasm exchange as infection is mainly by infected tubers.

## 1 Introduction

Yam (*Dioscorea* spp.) is an annual herbaceous vine with edible starchy tubers. Globally, it is the fourth most important tuber crop, while in Africa, it is second after cassava by value and production [1,2]. Tubers of domesticated yam provide an essential source of carbohydrate for over 60 million people in tropical and subtropical regions, with 95% of global production deriving from West Africa [3]. The white yam (*D. rotundata*), a native species to West Africa dominates the area of production, and the water yam (*D. alata*) the second most frequently cultivated species, has Asian origin.

Viruses belonging to at least five genera have been reported to infect yams around the world. Yam mild mosaic virus (YMMV) is the second most prevalent potyvirus affecting yams after Yam mosaic virus (YMV) [4,5]. The virus occurrence was reported from several yam producing countries in Africa [6,7], Asia and Oceania [4,8,9] and the Caribbean [10]. Initially, YMMV was described as yam virus 1 (YV1) [8] and *Dioscorea alata* virus (DaV) [7]. Mumford and Seal [4] classified YMMV as a distinct potyvirus, which was further confirmed by the serological and molecular characterization of isolates from Martinique, Colombia, and Brazil [[11], [12], [13]]. The virus spreads mainly through the vegetative propagation of tubers or vine cuttings sourced from the virus infected plants. YMMV transmission by the aphid, *Aphis craccivora* Koch, has also been reported [7], but the significance of the aphid vector in YMMV transmission has not been established.

The YMMV genome is about 9540 nucleotides (nt) in size encodes a large open reading frame (ORF), which cleaves into 11 functional proteins: P1, HC-Pro, P3, 6K1, CI, 6K2, NIa-VPg, NIa-Pro, Nib, PIPO and CP [10]. The coat protein (CP) gene of YMMV is 798 nt long and codes for a peptide comprising 266 amino acids (aa) with a mass of 30 kDa. The DAG motif that is present in the CP of most potyviruses ([14] has been shown to be important for aphid transmission [15].

This study was conducted to determine the YMMV diversity in Nigeria, the leading global producer of yam, and Ghana the second largest producer of yam, together, they account for 76.3% of global yam production [2]. Although YMMV is not a significant constraint to yam production in West African region, it is important to understand the dispersion and diversity of YMMV populations in these major yam producing countries. Potyviruses are subject to rapid evolution and associated high levels of molecular variability [10] that may lead to variants with higher virulence than those currently known [10]. Elucidation of YMMV diversity will enable the identification of targets for use in diagnostic tests that are urgently needed to certify virus-free yam planting materials in West Africa [3].

In this study, we determined the genetic diversity of 18 YMMV isolates by sequencing their complete CP encoding gene and the sequences were used for multiple sequence alignments and phylogenetic analysis together with 8 YMMV CP sequences available in the NCBI GenBank database.

## 2 Materials and methods

### 2.1 Plant material

The YMMV infected yam samples used for sequencing were collected during the surveys in the major yam producing areas across three agro-ecological zones of Nigeria and Ghana, Humid Forest (HF), Derived Savana (DS), and Southern-Guinea Savana (SGS), in 2012 and 2013. A total of 65 fields (31 and 34 in Ghana and Nigeria, respectively) in 2012, and 75 fields (34 and 41 in Ghana and Nigeria, respectively) in 2013 were surveyed. In each field, 30 plants were assessed for symptoms, and a representative set of samples were collected for virus testing and they were preserved in 30 mL glass vials containing Tris buffer, pH 8.0, with 2% (w/v) cetyl trimethyl ammonium bromide (CTAB). A total of 1530 leaf samples (both symptomatic and asymptomatic) were collected for virus indexing from both years. Samples were analyzed for YMMV using reverse transcriptase-polymerase chain reaction (RT-PCR) at the Virology and Molecular Diagnostic Unit at the International Institute of Tropical Agriculture (IITA), Ibadan, Nigeria.

### 2.2 Extraction of total nucleic acids

Total nucleic acids were extracted using the modified CTAB procedure described by Abarshi et al. [16]. Approximately 100 mg of leaf material was grounded in 1 mL CTAB buffer and 600 μL of the extract was transferred into a 1.5-ml tube and incubated at 60 °C for 10 min. Equal volume of phenol:chloroform: isoamyl alcohol (25:24:1) was added to the extract and then centrifuged at 12,000 *g* for 10 min. The supernatant was transferred to a new 1.5 ml tube, to which 300 μL of ice-cold iso-propanol was added and incubated at −20 °C for 60 min and then tubes were centrifuged at 12,000 *g* for 10 min to precipitate nucleic acids. The precipitated pellet was washed with 0.5 mL of 70% (v/v) ethanol, centrifuged for 5 min, and the pellet was air-dried at room temperature. The final pellet was suspended in 50 μL of sterile distilled water and stored at −20 °C until used. Total nucleic acid was quantified using a NanoDrop 2000 spectrophotometer (Thermo Scientific, UK) and nucleic acid integrity was analyzed by electrophoresing a 4 μL aliquot on a Tris-Acetate EDTA (TAE) agarose gel (Sigma, France) at 110 V for one h. Gels were stained with 0.5 μg/mL ethidium bromide and visualized under ultraviolet (UV 302 nm) light using a EZ Gel Imager (Bio-Rad, France).

### 2.3 YMMV detection using RT-PCR

RT-PCR was performed using a YMMV specific primer pair (YMMV-CP-Bam FP: 5′-CAGAGAGGATCCGCAAGTAAGGAACAGACATTTG -3′ and YMMV-CP-EcoRP: 5′ -TTGATCGAATTCCTAGATATTGCGCACTCCAAGAAG -3′) designed to amplify full-length CP (798 bp) segment. The RT-PCR was set up in 12.5 μL reactions containing 2 μL of total nucleic acid template (at 100 ng/μl), 0.2 μM of each primer, 0.2 mM of each dNTP, 1 U GoTaq DNA Polymerase (Promega, USA), 12 U M-MLV reverse transcriptase (Promega, USA) and 1x GoTaq Reaction Buffer (Promega, USA). Reactions were incubated in a thermocycler (PCR system 9700, Applied Biosystems, Singapore) set at 44 °C for 30 min and 95 °C for 5 min for one cycle, followed by a 35 cycles of amplification by denaturation at 95 °C for 30 s, primer annealing at 55 °C for 30 s and extension at 72 °C for 60 s; finally, one cycle of extension at 72 °C for 10 min. RT-PCR products were electrophoresed and visualized on a 1% (w/v) TAE agarose gel containing 0.5 μg/mL ethidium bromide under UV light using a EZ Gel Imager (Bio-Rad, France).

The PCR amplicons (798bp) were sequenced in both directions at the Iowa State University DNA Sequencing Facility (Ames, Iowa, USA). The nucleotide sequence data were analyzed using BioEdit and MEGA7 software [17,18] and the consensus sequences for each isolate were used for sequence similarity searches in the GenBank databases using the BLAST program [17] and amino acid sequences were deduced using Transeq [19]. Multiple sequence alignments were generated using CLUSTAL W [20], and pairwise distances were estimated using MEGA7 software. The 18 YMMV isolates sequenced in this study and eight reference YMMV CP sequences downloaded from the NCBI database (Table **S**1) were used for the phylogenetic analysis using the neighbor-joining method [21] using MEGA 7 software.

## 3 Results

**Fig. 1:**
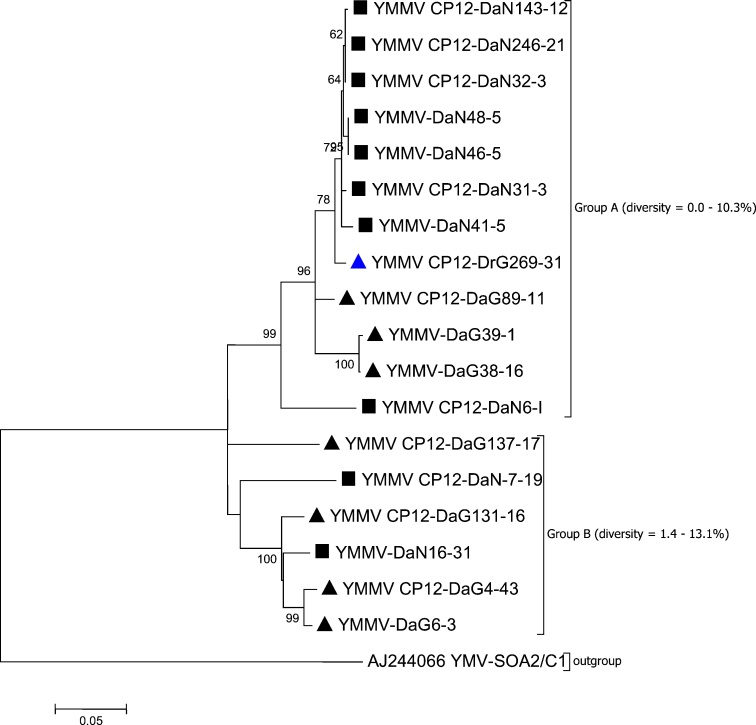
Cladogram based on sequences of the CP region of 18 YMMV isolates collected from *D. alata* in Nigeria (◼) and Ghana (▲) and from *D. rotundata* in Ghana (), using neighbour-joining method. The tree was generated using neighbour-joining method in MEGA7 with 1000 bootstrap replications, with branches <60% were collapsed. The scale bar represents the number of nucleotide substitutions per site.

The YMMV was detected in 39 (2.5%) of the 1530 samples tested from 18 (12.8%) of 140 sampled locations (Table S2). The YMMV CP gene from virus-positive isolates representing each of the 18 locations were sequenced for diversity analysis. The mean pairwise nucleotide (nt) diversity between the 18 YMMV sequences was 13.4%. The mean nt diversity between the isolates collected from Ghana was 11.4% at nt level and 4.9% at the amino acid (aa) level, whereas the mean nt and aa diversity was 7.4% and 1.8%, respectively, between the isolates from Nigeria. The sequence diversity was high among the YMMV isolates in Ghana compared with isolates in Nigeria. Except for a single isolate from Ghana (YMMV-CP12-DrG296−31; *D. rotundata*), all the YMMV isolates detected in this study were from *D. alata*. The phylogenetic analysis clustered YMMV isolates into two major groups, names as A and B (Fig. 1). Group A comprised 12 isolates (4 from Ghana and 8 from Nigeria), while Group B comprised six isolates (4 from Ghana and 2 from Nigeria). The nt diversity in Group A ranged from 0 to 10.3% and 1.4–13.1% in Group B (Fig. 1).

**Fig. 2:**
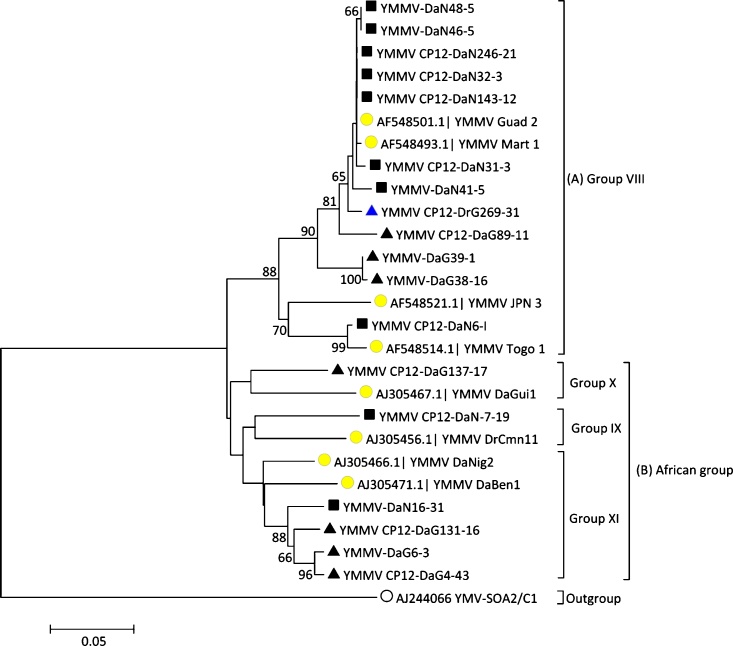
Cladogram based on full-length coat protein encoding nucleotide sequences of the 18 YMMV isolates from *D. alata* from Nigeria (◼) and Ghana (▲) and from *D. rotundata* in Ghana () in this study together with the YMMV reference isolates () from the NCBI GenBank. The tree was generated using neighbour-joining method in MEGA7 with 1000 bootstrap replications, with branches <60% were collapsed.. The percentage of trees in which the associated taxa clustered together is shown next to the branches. The scale bar represents the number of nucleotide substitutions per site.

The clustering of the 18 isolates (17 from *D. alata* and one from *D. rotundata*) did not correlate with their geographical origin (Fig. 2). Comparison of these isolates with reference YMMV sequences available in the NCBI GenBank clustered 18 YMMV isolates sequenced in this study into four monophyletic groups, VIII, IX, X and XI, as per the classification by Bousalem et al. [10] (Fig. 2). The YMMV isolates clustered in the group VIII have wide distribution and they were referred to as 'cosmopolitan group'. Whereas the YMMV isolates clustered in groups IX, X, and XI were mainly of Africa origin. The YMMV isolates of Group A were clustered with the monophyletic group VIII (cosmopolitan group), while those of Group B aligned to the three African monophyletic groups IX, X, and XI. Percent pairwise dissimilarity within the groups ranged from 5.0 to 18.1%, while the overall mean was 10.4 % (Table S3).

**Fig. 3:**
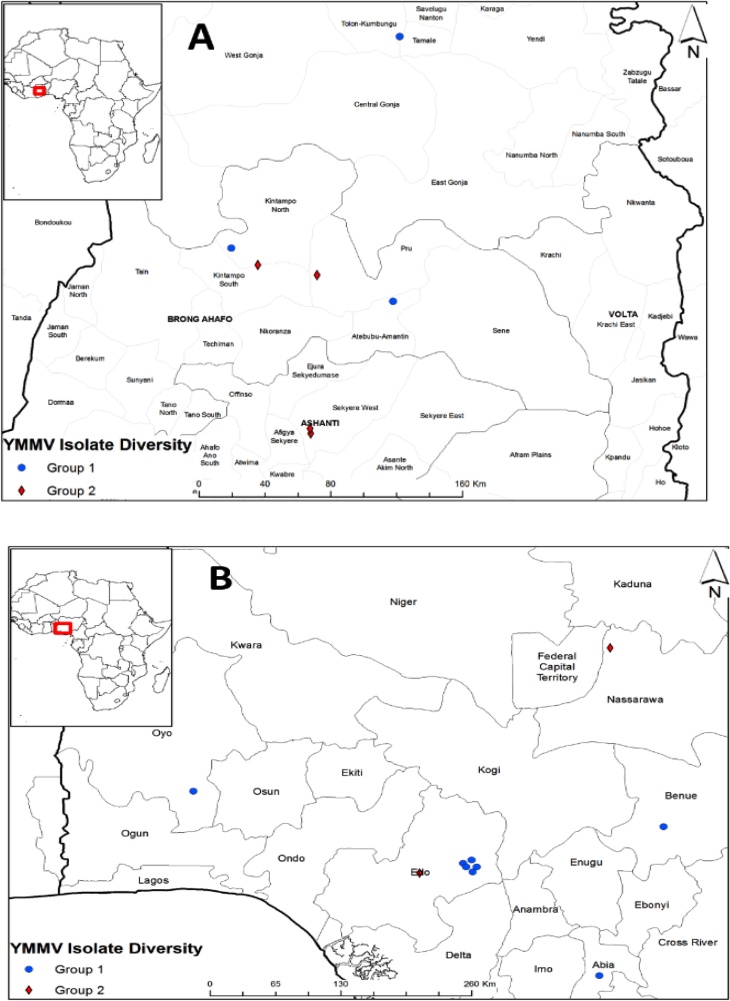
Geographic distribution of Group A (Cosmopolitan group; ) and Group B (African group; ) YMMV isolates in Ghana (A) and Nigeria (B).

The geographic distribution of YMMV isolates used for diversity studies in Ghana and Nigeria in the different agro-ecologies is shown in Fig. 3. YMMV isolates of Nigeria affiliated to Group A (cosmopolitan group) were detected in HF and DS, while Group B (African origin) was detected in DS and SGS. The YMMV isolates of Ghana affiliated to the Group A were detected in DS and SGS, and the Group B was detected in HF and DS zones. The YMMV isolates in Group A clustered with isolates from Togo, Japan, Martinique, and Guadeloupe, while Group B clustered with isolates from Benin, Nigeria, Guinea, and Cameroon.

## 4 Discussion

Genetic diversity of YMMV in Ghana and Nigeria was investigated using CP nucleotide sequences of 18 isolates collected during surveys conducted in 2012–13. Multiple sequence analysis indicated nucleotide identities within these isolates were >80%, which is higher than the suggested ≤76–77% nucleotide identity for species demarcation for the potyvirus CP gene [22]. The clustering pattern of the 18 isolates did not correlate with the geographical origin of the samples. Most isolates from Nigeria affiliated to phylogenetic Group VIII (Group A), whereas those from Ghana were clustered to Groups A and B. Although the cause of this variability was not assessed, studies, such as those by Fereres et al. [23] and Hammond-Kosack and Jones [24] have shown that aphid transmission of viruses may lead to genetic variability in the pathogen, due to exposure to multiple adaption stresses associated with the feeding of these vectors on a diversity of host plants.

Comparison of the CP sequences of 18 isolates obtained in this study with eight YMMV reference isolates from the NCBI GenBank showed that those from Ghana and Nigeria clustered with isolates from other countries corresponding to the four of the eleven monophyletic groups proposed for YMMV by Bousalem et al. [10]. For example, YMMV isolates Guadeloupe 2, Mart 1, JPN 3 and Togo 1 from Guadeloupe, Martinique, Japan, and Togo, respectively, which are members of YMMV monophyletic group VIII (the cosmopolitan group), clustered with isolates in Group A, while isolates DrGui1, DaNig2 and DaBen1 and DrCmn 11 from Guinea, Nigeria, Benin, and Cameroon, respectively, clustered with isolates in Group B, which is the African group that comprises monophyletic groups IX, X, and XI. YMMV CP12-DaN6-1, an isolate from Nigeria, shared 99% nt identity to Togo 1 GenBank Accession AF548514) suggesting possibility of seed-borne spread of YMMV in West Africa.

Although this study revealed a low prevalence of YMMV in Nigeria and Ghana, sequence diversity showed evidence of regional and international distribution of viruses through planting material exchange. Nigeria and Ghana are the largest producers and exporters of yam, respectively, and in the event of YMMV strains/isolates with higher virulence, the transboundary proliferation of the virus could impair yam production and productivity in the two countries and more widely in West Africa. The quarantine monitoring of germplasm needs to be strengthened to mitigate this risk, coupled with improvements to the production and distribution of clean planting for seed and ware yam production. The sequences generated in the study will facilitate the development of field-based diagnostic tools necessary for virus indexing and seed certification as it was achieved with YMV [[25], [26], [27]].

## 5 Conclusions

This study revealed a low prevalence of YMMV in Nigeria and Ghana. YMMV was mainly detected in *D. alata*, an Asiatic yam introduced to West Africa in the 16th century. The YMMV sequence diversity indicated occurrence of the isolates corresponding to the ‘cosmopolitan group’ and the ‘Africa group’ but there was no correlation with the geographic location of the isolate, which suggests the potential seed-borne spread through the exchange of virus-infected tubers within the region. Data from this study will be useful for the development of specific and sensitive diagnostic tools for YMMV for seed certification and quarantine monitoring.

## Declaration of Competing Interest

The authors declare that they have no known competing financial interests or personal relationships that could have appeared to influence the work reported in this paper.
